# Toward a priori noise characterization for real-time edge-aware denoising in fluoroscopic devices

**DOI:** 10.1186/s12938-021-00874-8

**Published:** 2021-04-07

**Authors:** Emilio Andreozzi, Antonio Fratini, Daniele Esposito, Mario Cesarelli, Paolo Bifulco

**Affiliations:** 1 Department of Electrical Engineering and Information Technology, University of Naples Federico II, Via Claudio 21, 80125 Naples, Italy; 2 Biomedical Engineering, School of Life and Health Sciences, Aston University, Birmingham, B4 7ET UK

**Keywords:** Quantum noise, Poisson noise, X-ray imaging, Fluoroscopy, Noise estimation, Noise characterization, Edge-aware denoising, Real-time denoising

## Abstract

**Background:**

Low-dose X-ray images have become increasingly popular in the last decades, due to the need to guarantee the lowest reasonable patient’s exposure. Dose reduction causes a substantial increase of quantum noise, which needs to be suitably suppressed. In particular, real-time denoising is required to support common interventional fluoroscopy procedures. The knowledge of noise statistics provides precious information that helps to improve denoising performances, thus making noise estimation a crucial task for effective denoising strategies. Noise statistics depend on different factors, but are mainly influenced by the X-ray tube settings, which may vary even within the same procedure. This complicates real-time denoising, because noise estimation should be repeated after any changes in tube settings, which would be hardly feasible in practice. This work investigates the feasibility of an a priori characterization of noise for a single fluoroscopic device, which would obviate the need for inferring noise statics prior to each new images acquisition. The noise estimation algorithm used in this study was tested in silico to assess its accuracy and reliability. Then, real sequences were acquired by imaging two different X-ray phantoms via a commercial fluoroscopic device at various X-ray tube settings. Finally, noise estimation was performed to assess the matching of noise statistics inferred from two different sequences, acquired independently in the same operating conditions.

**Results:**

The noise estimation algorithm proved capable of retrieving noise statistics, regardless of the particular imaged scene, also achieving good results even by using only 10 frames (mean percentage error lower than 2%). The tests performed on the real fluoroscopic sequences confirmed that the estimated noise statistics are independent of the particular informational content of the scene from which they have been inferred, as they turned out to be consistent in sequences of the two different phantoms acquired independently with the same X-ray tube settings.

**Conclusions:**

The encouraging results suggest that an a priori characterization of noise for a single fluoroscopic device is feasible and could improve the actual implementation of real-time denoising strategies that take advantage of noise statistics to improve the trade-off between noise reduction and details preservation.

**Supplementary Information:**

The online version contains supplementary material available at 10.1186/s12938-021-00874-8.

## Background

Fluoroscopy is a medical imaging modality that provides continuous, real-time X-ray screening of patient’s organs and of various radiopaque objects involved in surgical procedures (e.g., surgical instruments, catheters, wire-guides, prosthetic implants, implanted devices), which make it an invaluable tool for image-guided procedures in surgery [1, 2], as well as in diagnosis [3–5] and therapy [6]. However, its use in clinical practice should always be carefully evaluated, as X-rays are ionizing radiations that may cause serious damages to human tissues and organs [7–10], and that is why the rigorous monitoring of the X-ray dose delivered to the patients and to the exposed medical staff has gained progressively more attention in the last decades, also being subject to formal regulations from national and international health organizations [11–13]. The X-ray dose depends on a number of parameters and conditions, such as the X-ray tube settings (tube current and voltage), the exposure time, the distance between the X-ray source and the irradiated tissue, the additional filtration, the number of anti-scatter grids [14]. Generally, most of these parameters are selected to optimize determined features of the imaged scene, thus, only the tube current and, sometimes, the exposure time can be modified to reduce the overall dose delivered to the patient. As an example, a common practice to limit the overall exposure time in fluoroscopy during surgical procedures is to turn off the X-ray source periodically and/or to use pulsed protocols, which place a limitation on frame rate though [14]. However, the exposure times are still very long and unpredictable in interventional fluoroscopy [10, 15], as they depend on the particular needs of the surgeon in each procedure.

In practice, the dose is mainly limited by reducing the tube current, which implies a reduction of the X-ray radiation intensity, i.e., the number of X-ray photons that reach the detector. This low photons availability gives rise to a signal-dependent, Poisson-distributed noise, usually referred to as “quantum noise” or “Poisson noise” [16]. The signal-to-noise ratio (SNR) of quantum noise decreases as the square root of the mean luminance, which means that the lower the dose, the lower the image quality [16]. Moreover, quantum noise is inherent to the image formation process and cannot be avoided or even limited by improving detectors technology, thus requiring the application of proper denoising strategies in the digital domain [16].

Simple smoothing filters usually do not achieve acceptable results, as they introduce significant blurring effects (in space and time), thus accomplishing noise reduction to the detriment of fine image details (e.g., edges, textures, etc.). As for many denoising approaches devised for AWGN, the knowledge of noise statistics provides precious information that helps to improve the denoising performances [16–29]. While scientific literature is rich in approaches for AWGN estimation, much lower effort has been devoted to Poisson noise [16, 26–28, 30–34], even though it is by far the dominant noise source in low-dose X-ray images [16, 35–37], as well as in other low-light images, e.g., night photography, fluorescence microscopy, astronomical imaging.

Quantum noise estimation could be used, e.g., to allow denoising algorithms discriminate between the noisy pixels to be filtered and those lying on the edges, which need to be preserved as much as possible to maintain the image details. This is the case, as an example, for the noise variance conditioned average (NVCA) algorithm [16–21]. This denoising strategy is based on a conditioned moving average filter that acts on a determined spatio-temporal neighborhood by including in the average computation only those pixels whose difference in luminance with the central pixel is lower than a multiple of the local noise standard deviation (SD). NVCA derives local estimates of noise SD by assuming a linear relationship between the variance and the expected value of the noise (Poisson–Gaussian noise model), whose slope and intercept, referred to as noise parameters, must be determined prior to the filtering operation. Other approaches involves the use of variance stabilizing transformations [27, 38–40], the most common of which is the generalized Anscombe transform [41, 42]. This point-wise operation transforms a Poisson–Gaussian distribution in a practically Gaussian distribution with unit variance, thus allowing the use of any AWGN denoising scheme also for Poisson–Gaussian noise. However, the generalized Anscombe transform also requires the a priori knowledge of noise parameters.

Essentially, the denoising approaches that make direct use of Poisson statistics, as well as those based on the combination of generalized Anscombe transform with AWGN denoising schemes, both require the noise parameters to be accurately estimated from noisy images prior to their actual processing, in order to achieve a reasonable trade-off between noise reduction and edge preservation, especially in images that are heavily affected by noise (e.g., low-dose X-ray images). While it is not usually a major concern in offline implementations, it could be a serious limitation in real-time operation, which undoubtedly represents the most appealing application of fluoroscopic sequence denoising. Indeed, during an image-guided procedure the variations of tube settings and of detector gain would modulate the statistics of quantum noise. Hence, the noise parameters estimation should be repeated ideally after any change in X-ray tube settings to ensure the highest denoising performances, but this is hardly feasible in practice.

This study aims to test the hypothesis that the noise parameters mostly depend on the X-ray tube settings and presents a feasibility analysis of an a priori noise parameters characterization. Indeed, this approach would obviate the need for inferring noise statics prior to each new image sequence acquisition, thus enabling the effective real-time operation of edge-aware denoising strategies that take advantage of noise statistics to improve the image quality in fluoroscopic sequences. The influence of the X-ray tube settings on the noise parameters has never been investigated before in literature, although this greatly affects real applications. The study also suggests a practical approach to provide denoising algorithms with accurate noise estimates to ensure the highest performances in real-time.

The noise estimation algorithm has already been used in previous publications about the NVCA denoiser [16, 17, 20, 21], but its performances have never been assessed thoroughly. In this study, the algorithm was first tested in silico on several synthetic fluoroscopic sequences, which were corrupted by different levels of simulated mixed Poisson–Gaussian noise. The ability of the algorithm to retrieve the noise parameters with reasonable accuracy was assessed by varying the number and distribution of grey levels within the designed sequences, as well as the number of frames exploited for noise estimation. Afterward, real fluoroscopic sequences were acquired by imaging two different X-ray phantoms via the same commercial fluoroscopic device with various X-ray tube settings. Then, the matching of noise parameters was assessed between data acquired independently in the same operating conditions, as it would support the prospect of pre-calibrating the noise parameters at many different tube settings and using them directly in real-time denoising of new fluoroscopic sequences acquired in the same conditions.

## Results

### Performance assessment on synthetic sequences

**Fig. 1:**
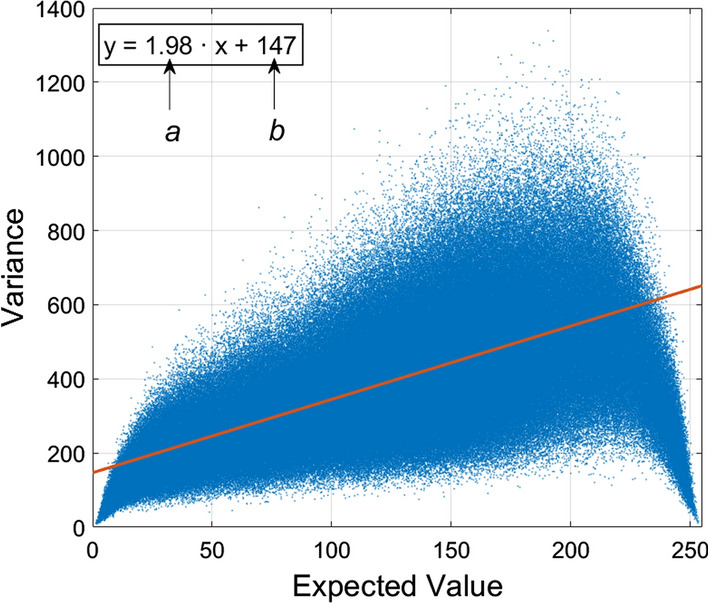
An example of a typical measured EVaR with its linear regression and the estimated noise parameters (**a**, **b**)

Figure 1 shows an example of a typical measured EVaR with its linear regression. In Additional file 1: Table S1 (available in the Additional file 1, along with Tables S2, S3, S4) the noise parameters estimates extracted from all the 14 synthetic sequences with variable number of grey levels are reported. For each sequence, the parameters were subdivided by the corresponding noise level and the number of frames used for noise estimation. Additional file 1: Table S2 outlines the relative estimation errors, except for the errors related to null nominal values of parameter *b* (i.e., noise levels 1 to 3), which were reported as absolute errors and highlighted in blue. A substantial difference was observed in the estimation errors obtained in sequences 1–7 and sequences 8–14, which turned out to be on average consistently higher than those of the former. Mean and standard deviation (SD) of the estimation errors are reported in Table 1.

**Table 1:** Mean and standard deviation of the noise parameters estimation errors

Parameter error	Sequences	Errors on noise parameters estimates							
		*F* = 100		*F* = 50		*F* = 25		*F* = 10	
		Mean	SD	Mean	SD	Mean	SD	Mean	SD
${a}_{er{r}_{p}}$	1–7	− 0.0025	0.0056	− 0.0042	0.0065	− 0.0062	0.0105	− 0.0126	0.0176
	8–14	− 0.0226	0.0422	− 0.0280	0.0503	− 0.0467	0.0825	− 0.1078	0.1643
${b}_{er{r}_{p}}$	1–7	0.0051	0.0083	0.0068	0.0094	0.0115	0.0144	0.0175	0.0235
	8–14	0.0362	0.0724	0.0458	0.0920	0.0751	0.1610	0.1686	0.2793
${b}_{er{r}_{abs}}$	1–7	0.2755	0.5082	0.3763	0.7232	0.6742	0.8471	1.5837	2.1418
	8–14	3.2800	6.7123	4.0795	7.5838	7.5403	16.832	14.885	30.1688

The statistics are computed for sequences 1–7 and 8–14 separately, and for each considered number of available frames (*F*)

Since noise estimation serves as a support for improving noise suppression performances, its efficacy should be rather assessed by analyzing the effect of estimation errors on the final denoising results. To this aim, the noisy synthetic test sequences described in the “Materials and methods” section, and depicted in Fig. 10, were filtered via the NVCA algorithm by using the most inaccurate noise parameters estimates, so as to identify the worst cases from the denoising point of view. Then, the worst results obtained for estimates extracted by using 25 and 10 frames, from sequences 1–7 and 8–14, were identified according to measures of dissimilarity between the sequences filtered with inaccurate noise parameters, referred to as the sub-optimal filtered sequences, and the sequence filtered with the actual noise parameters, referred to as the optimal filtered sequence. Two well-established image quality assessment indices were adopted, namely the mean squared error (MSE), which is a global measure of dissimilarity between images, and the full width at half maximum (FWHM) of the edge spread function, which is a no-reference local measure of edge sharpness [20]. As a first dissimilarity measure to quantify the global deviation from the optimal denoising result, the MSE between the sub-optimal and the optimal filtered sequences was computed. However, MSE is known to have high sensitivity to the overall image noise, but poor sensitivity to edge blurring effects, especially in noisy conditions like those encountered in low-dose fluoroscopy [22]. Since the edge-awareness is a major concern of medical image denoising, the local loss of edge sharpness, due to the noise parameters estimation errors, was considered as a further measure of dissimilarity, and was evaluated by estimating the Δ FWHM, that is the difference in FWHM between the sub-optimal and the optimal filtered sequences. The quantitative results of this analysis are summarized in Tables 2 and 3, where it could be noticed that the worst results were always obtained by using the parameters extracted from the sequences 8–14, which were also those affected by the highest estimation errors.

**Table 2:** Results of the denoising performance analysis on the sequence with the moving rectangle

Sequences	*a*	*b*	*F*	${a}_{est}$	${b}_{est}$	$MSE\left({S}_{filt},{S}_{fil{t}_{err}}\right)$	$\Delta FWHM$
1–7	2	0	25	1.979	3.007	0.090284	0.001851
	2	0	10	1.937	7.186	0.199485	0.002836
8–14	2	0	25	1.425	74.866	2.276551	0.041098
	2	0	10	0.984	131.379	5.324343	0.256335

The dissimilarity scores between the sub-optimal and optimal filtered sequences are reported, along with the corresponding noise parameters estimates, the number of frames (*F*) used in the estimation, and the actual noise parameters values

**Table 3:** Results of the denoising performance analysis on the sequence with the moving circle

Sequences	*a*	*b*	*F*	${a}_{est}$	${b}_{est}$	$MSE\left({S}_{filt},{S}_{fil{t}_{err}}\right)$	$\Delta FWHM$
1–7	2	0	25	1.979	3.007	0.017391	0.002190
	2	0	10	1.937	7.186	0.082492	0.005973
8–14	2	0	25	1.425	74.866	1.957688	0.116500
	2	0	10	0.984	131.379	4.702226	0.422549

The dissimilarity scores between the sub-optimal and optimal filtered sequences are reported, along with the corresponding noise parameters estimates, the number of frames (F) used in the estimation, and the actual noise parameters values

**Fig. 2:**
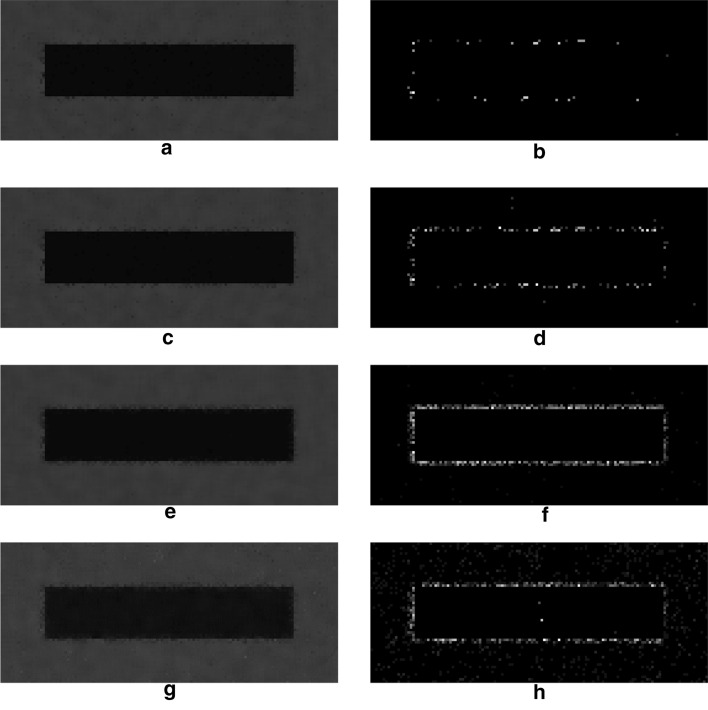
Synthetic sequences with the moving rectangle filtered via the NVCA algorithm by considering the noise parameters estimates reported in Table 2. The images in each row were obtained by using the noise parameters in the corresponding row of Table 2. On the first column the end frames of the sub-optimal filtered sequences were depicted, while the differences of the same images with the end frame of the optimal filtered sequence were reported on the second column

**Fig. 3:**
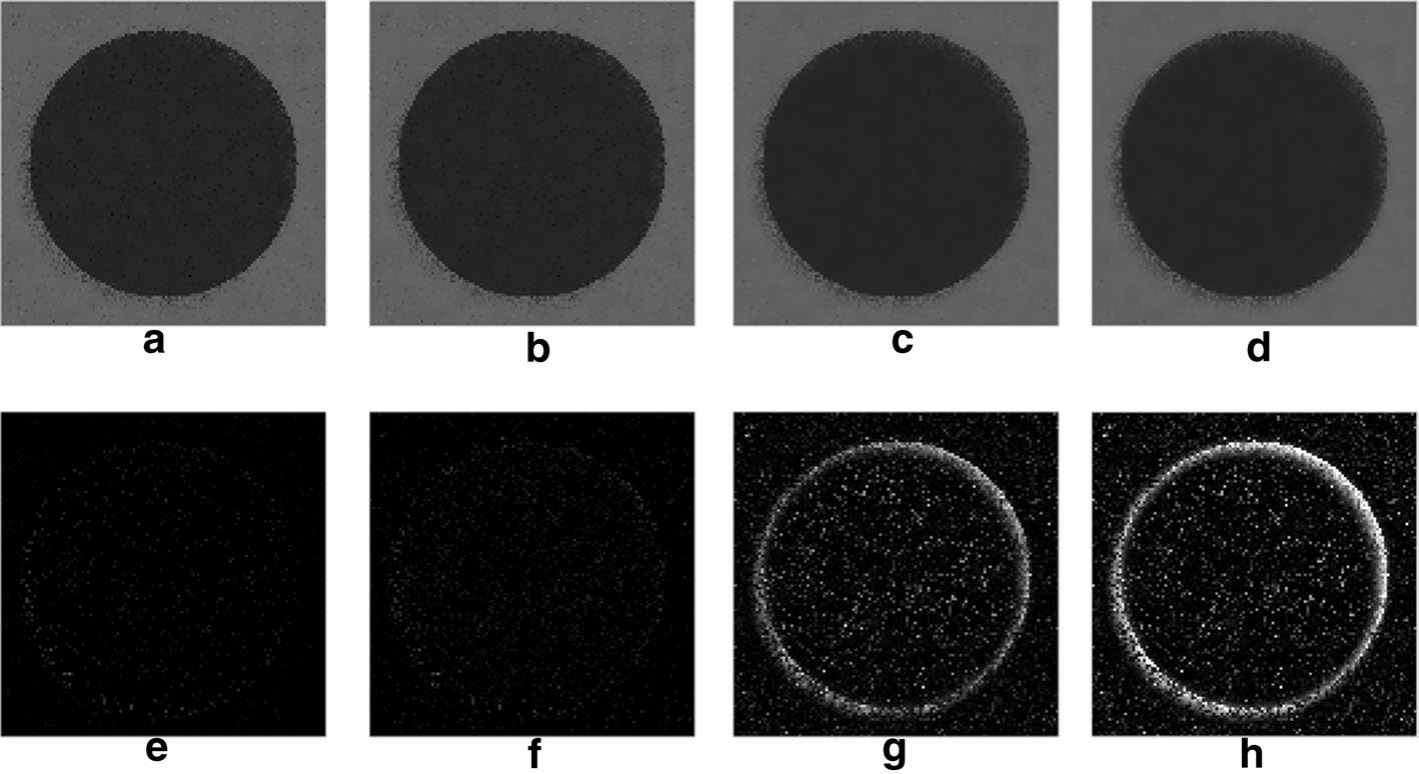
Synthetic sequences with the moving circle filtered via the NVCA algorithm by considering the noise parameters estimates reported in Table 3. The images in each column were obtained by using the noise parameters in the corresponding row of Table 3. On the first row the end frames of the sub-optimal filtered sequences were depicted, while the differences of the same images with the end frame of the optimal filtered sequence were reported on the second row

Figures 2 and 3 depict the sub-optimal filtered sequences, as well as the corresponding image differences with the optimal filtered sequence, where it can be observed that the pixels with the highest differences in luminance are almost all distributed in the edges neighborhood, which is consistent with the measured increase in Δ FWHM.

However, it can be assessed by visual inspection that the sub-optimal results shown in Figs. 2a–c and 3a, b are very similar the corresponding optimal results shown in Fig. 10c and d, respectively.

The results of the noise parameters extraction from the four synthetic sequences designed via the X-ray simulator are reported in Additional file 1: Tables S3 and S4. The mean and SD of relative errors, outlined in Table 4, turned out to be almost comparable with those obtained in the 14 synthetic sequences, thus proving that the presence of clinically relevant structures does not alter the estimates accuracy, which, more generally, is not influenced by the particular informational content of the scene.

**Table 4:** Mean and standard deviation of the errors on noise parameters estimated in the sequences designed via the X-ray simulator by using 25 frames

Parameter error	Errors on noise parameters estimates	
	*F* = 25	
	Mean	SD
${a}_{er{r}_{p}}$	− 0.0135	0.0114
${b}_{er{r}_{p}}$	0.0226	0.0244
${b}_{er{r}_{abs}}$	1.0811	1.3120

### Noise estimation in real sequences

**Fig. 4:**
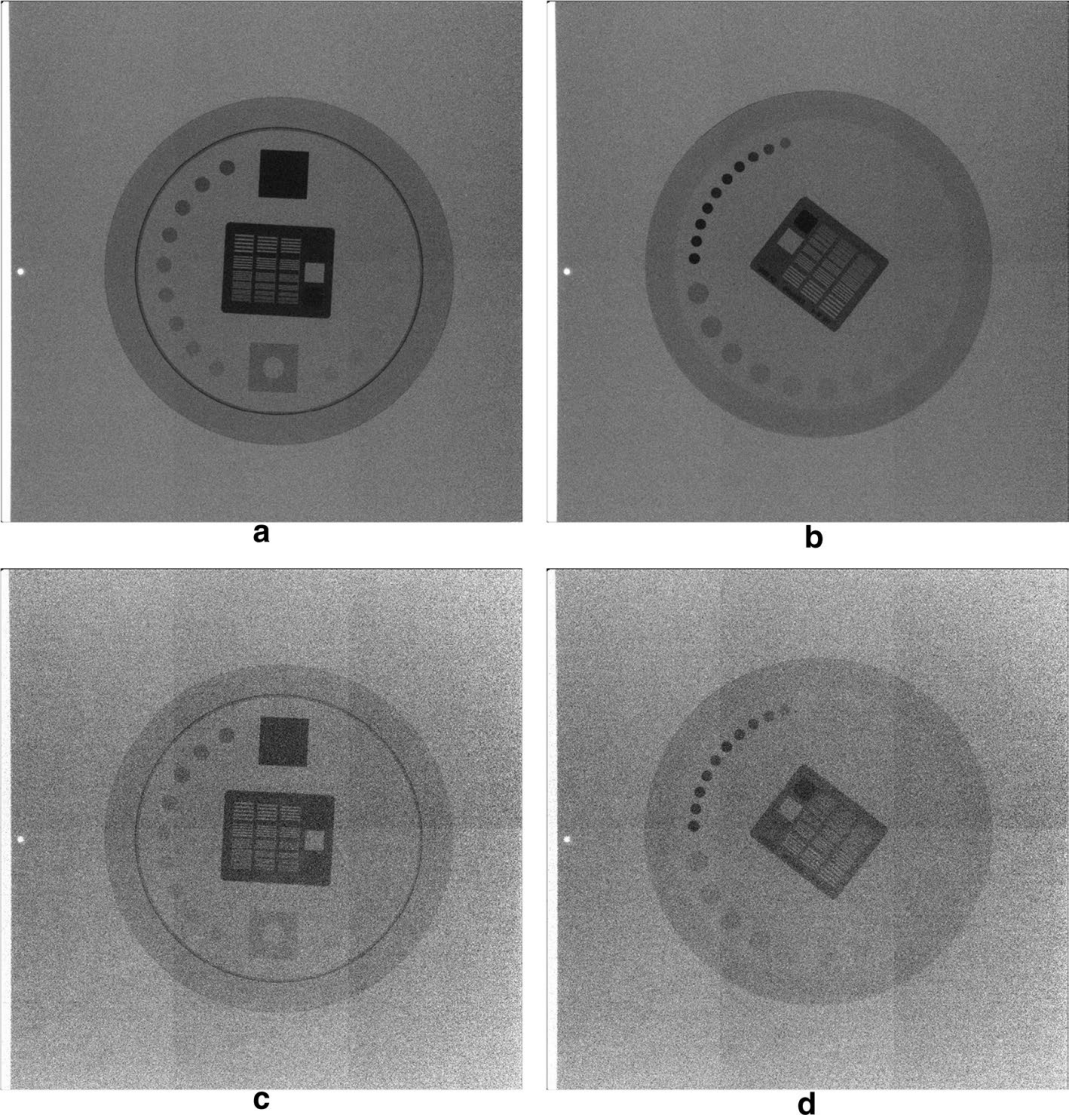
Static frames from the real fluoroscopic sequences. The frames shown in the first and second rows were acquired at 50 mA and 10 mA (40 kVp), respectively. The frames in the left column depict the TOR-18FG phantom, while those in the right column depict the TOR-CDR

Figure 4 shows four frames of the real fluoroscopic sequences, acquired as described in paragraph 5 of the “Materials and methods” section. In particular, the frames in the left column depict the TOR-18FG phantom, while the ones in the right column refer to the TOR-CDR phantom.

The frames in the first row were acquired with X-ray tube setting #5 (40 kVp, 50 mA), while those in the second row with setting #1 (40 kVp, 10 mA). Due to the very low tube currents involved, the original images turned out to be too dark for practical visualization, as indeed the luminance values were confined within very narrow ranges in the lower part of the representation interval. For this reason, the images in Fig. 4 have been processed with a full-scale histogram stretch, disregarding the grey levels of the few lightest pixels in the leftmost part of the images. This processing obviously altered the mean luminance, which was originally much lower in the images acquired at 10 mA compared to those acquired at 50 mA, but made the noise much more visible, allowing easier comprehension of the effect of X-ray tube current reduction on the SNR of the images.

The noise parameters extracted from the real fluoroscopic sequences are reported in Table 5, along with the relative errors of the parameters retrieved from TOR-CDR sequences with respect to those obtained from the TOR-18FG sequences for corresponding X-ray tube settings.

**Table 5:** Noise parameters estimates retrieved from the real fluoroscopic sequences, with relative errors on single parameters extracted from TOR-CDR sequences with respect to TOR-18FG ones for each tube setting

kVp	mA	TOR-18FG		TOR-CDR		Errors	
		$a$	$b$	$a$	$b$	${a}_{err}$	${b}_{err}$
40	10	7.15091	123.033	7.20909	125.312	0.00814	0.01852
40	20	4.37212	401.807	4.32512	384.611	− 0.01075	− 0.04280
40	30	3.45425	522.778	3.45683	526.265	0.00075	0.00667
40	40	2.99311	615.161	2.95042	633.000	− 0.01426	0.02900
40	50	2.66481	702.262	2.65917	704.725	− 0.00212	0.00351

**Fig. 5:**
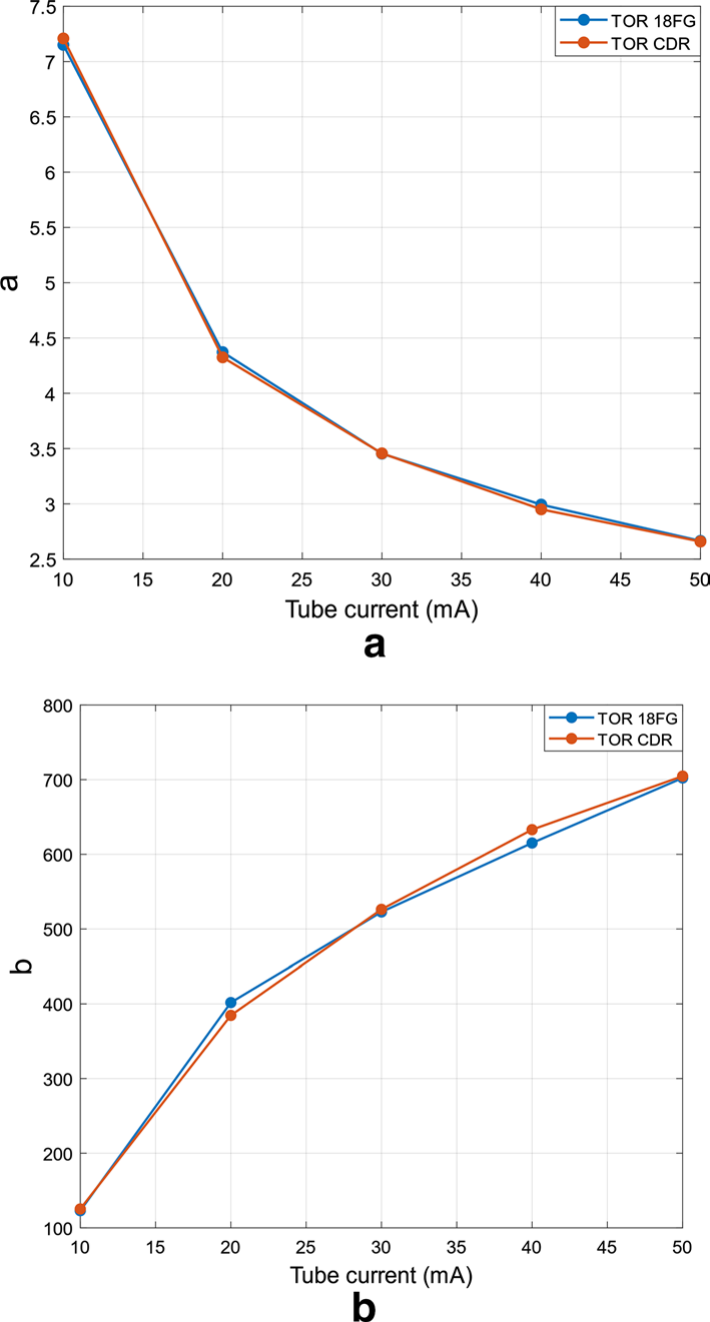
Noise parameters estimated from real fluoroscopic sequences. Static frames from the real fluoroscopic sequences. The frames shown in the first and second rows were acquired at 50 mA and 10 mA (40 kVp), respectively. The frames in the left column depict the TOR-18FG phantom, while those in the right column depict the TOR-CDR

The relative errors (mean and SD) were -0.36% ± 0.90% and 0.30% ± 2.8%, for parameters *a* and *b*, respectively, and turned out to be comparable to those obtained in the analyses of the performances of the noise parameters estimation algorithm. The noise parameters extracted from the two phantoms sequences are also plotted in Fig. 5, where it can be verified that their trends with the tube current are very similar.

## Discussion

This study investigated the feasibility of an a priori noise characterization at different X-ray tube settings for a single fluoroscopic device, which would obviate the need for inferring noise statics prior to each new image sequence acquisition, in order to enable the implementation of real-time algorithms that exploit the a priori knowledge of noise statistics to provide an effective, edge-aware denoising. To this aim, first the accuracies of the noise parameters provided by the considered noise estimation algorithm were assessed to ascertain their reliability. In particular, a first set of 7 synthetic sequences with increasing number of grey levels equally spaced in a 128-wide interval, and a further set of 7 synthetic sequences with 8 grey levels equally spaced in intervals of decreasing width were designed via software and, then, corrupted by six different levels of simulated Poisson–Gaussian noise, for a total of 84 noisy sequences. The noise parameters were extracted from each sequence, at each distinct noise level by considering four different numbers of available frames (i.e., 100, 50, 25, 10). The algorithm achieved very low estimation errors in the first seven sequences, while performing substantially worse on the last seven sequences. Indeed, it is worth noting that, even with only 10 turned out to exceed these value, even for estimations performed by using the maximum number of available frames. Furthermore, it could be assessed by visual inspection that, even the worst errors achieved in the sequences 1–7, produced sub-optimal filtered sequences which were very similar to the optimal one, as opposed to the sub-optimal sequences produced by the estimates from sequences 8–14, which clearly showed edge blurring (confirmed by increases in Δ FWHM). These results clarify that ensuring a reasonable contrast in the test sequences to be used for noise characterization is mandatory to achieve reliable estimates. Moreover, the very small number of frames required by the noise estimation algorithm to achieve a reasonable accuracy allows for its application also to very short static scenes. The algorithm accomplished comparable performances also on four synthetic sequences designed via an X-ray simulator to include realistic medical information, thus proving that the presence of clinically relevant structures does not alter the performances of the noise estimation algorithm.

Once accuracy and reliability of the noise parameters estimation had been assessed, the algorithm was applied to the real fluoroscopic sequences acquired by imaging two commercial X-ray phantoms with different tube settings. The noise parameters extracted from pairs of sequences acquired independently with the same tube settings turned out to be comparable, as the mean relative errors turned out to be less than 1%.

## Conclusions

The noise estimation algorithm considered in this study proved reliable in extracting noise parameters estimates with a reasonable accuracy, even from very short static scenes of only 10 frames. The tests performed on the real fluoroscopic sequences confirmed that the estimated noise parameters are independent of the particular informational content of the scene from which they have been extracted, as they turned out to be consistent in sequences acquired independently with the same X-ray tube settings. To the best of our knowledge, this is the first attempt to pre-characterize the noise of a single fluoroscopic device at different operating conditions, to obviate the need to repeat noise estimation after any change in X-ray tube settings. Moreover, it is also the first time that the trends of Poisson–Gaussian noise parameters with the X-ray tube settings are reported in literature. The encouraging results of this study suggest that an a priori characterization of noise for a single fluoroscopic device is feasible and could support the actual implementation of real-time edge-aware denoising strategies that take advantage of noise statistics to improve the trade-off between noise reduction and details preservation. Future studies could focus on a further characterization of noise, e.g., on an extended grid of X-ray tube settings (mA, kVp), also evaluating the possibility of obtaining part of these estimates via interpolation, as well as on an hardware implementation of the proposed approach, to directly assess its performances in real-time denoising of low-dose fluoroscopic sequences.

## Materials and methods

### Noise model

In an X-ray system, the number of photons that emerge from a patient and reach a single pixel of the detector plane can be modeled by a temporally stochastic Poisson process [16, 36, 37, 43], whose probability density function (pdf) is described in Eq. (1):

1 $$p\left(n\right)=\frac{{\uplambda }^{n}}{n!}{e}^{-\uplambda },$$

where $\uplambda$ is the expected photon count. Simple calculations allow deriving a very important feature of Poisson distribution, namely the expected value – variance relationship (EVaR), which is reported in Eq. (2):

2 $${\upsigma }_{p}^{2}\left({\upmu }_{p}\right)={\upmu }_{p}.$$

Therefore, the variance of the number of photons that reach a single pixel is equal to the expected photon count. However, in practice, the information carried by this random process occurring at a single detector pixel is usually coded in a digital image, and particularly in the grey level of the corresponding image pixel, which is proportional to the actual photon count, thus being characterized by a modified EVaR, as reported in Eq. (3):

3 $${\mathrm{g}\left(\uplambda \right)=a\cdot \mathrm{p}\left(\uplambda \right) \to \sigma }_{g}^{2}\left({\mu }_{g}\right)=a\cdot {\mu }_{g},$$

where *g* is the grey level of the digital image pixel corresponding to the detector pixel that is reached by a number of photons described by *p*, and *a* is the coefficient of proportionality between *g* and *p*, also known as “detector gain”. The EVaR clarify the signal-dependent nature of quantum noise (heteroscedasticity), which, unlike the well-known AWGN, cannot be characterized by a single, global noise variance estimate (homoscedasticity), but rather requires the estimation of the detector gain, in order to be able to estimate the local, signal-dependent noise variance from the local mean luminance.

X-ray images are also affected by other sources of noise that are usually modeled as AWGN, hence, they introduce a constant noise floor, i.e., a constant contribution to the noise variance, which can be included in the noise model, as shown in Eq. (4):

4 $${\sigma }_{g}^{2}\left({\mu }_{g}\right)=a\cdot {\mu }_{g} + b,$$

where $b$ corresponds to the variance of the AWGN component. This model is known as Poisson–Gaussian mixture and has been used in various denoising approaches devised for low-intensity images [16–24, 26, 32–34, 42]. However, it requires the knowledge of the EVaR parameters, referred to as noise parameters, which are generally unknown and, thus, need to be estimated from the X-ray images.

### Noise parameters estimation

The algorithm analyzed in this work infers the statistics of noise by taking advantage of the temporal dimension that is available in image sequences, such as those acquired in fluoroscopy. This approach can be applied only to static scenes, as it assumes the ideal, noiseless luminance of each pixel to be constant in time and ascribes all its fluctuations to the noise. Based on this assumption, the algorithm first calculates the sample mean and variance for each pixel along the temporal dimension, which describe the EVaR of the noise, and, then, it performs a linear regression to estimate the slope (*a*) and intercept (*b*) of the EVaR, i.e., the noise parameters. The number of frames available for noise characterization (i.e., the length of the static scene extracted from the fluoroscopic sequence of interest) poses a limitation on the actual number of observations of the random processes that describe each pixel luminance. This results in a certain variability of the variance values corresponding to the same mean value. This issue has been addressed in the performance analysis presented in this work, by evaluating the accuracy of noise parameters estimation also as a function of the number of available frames.

### Synthetic sequences design

#### Static sequences with variable number of grey levels

**Fig. 6:**
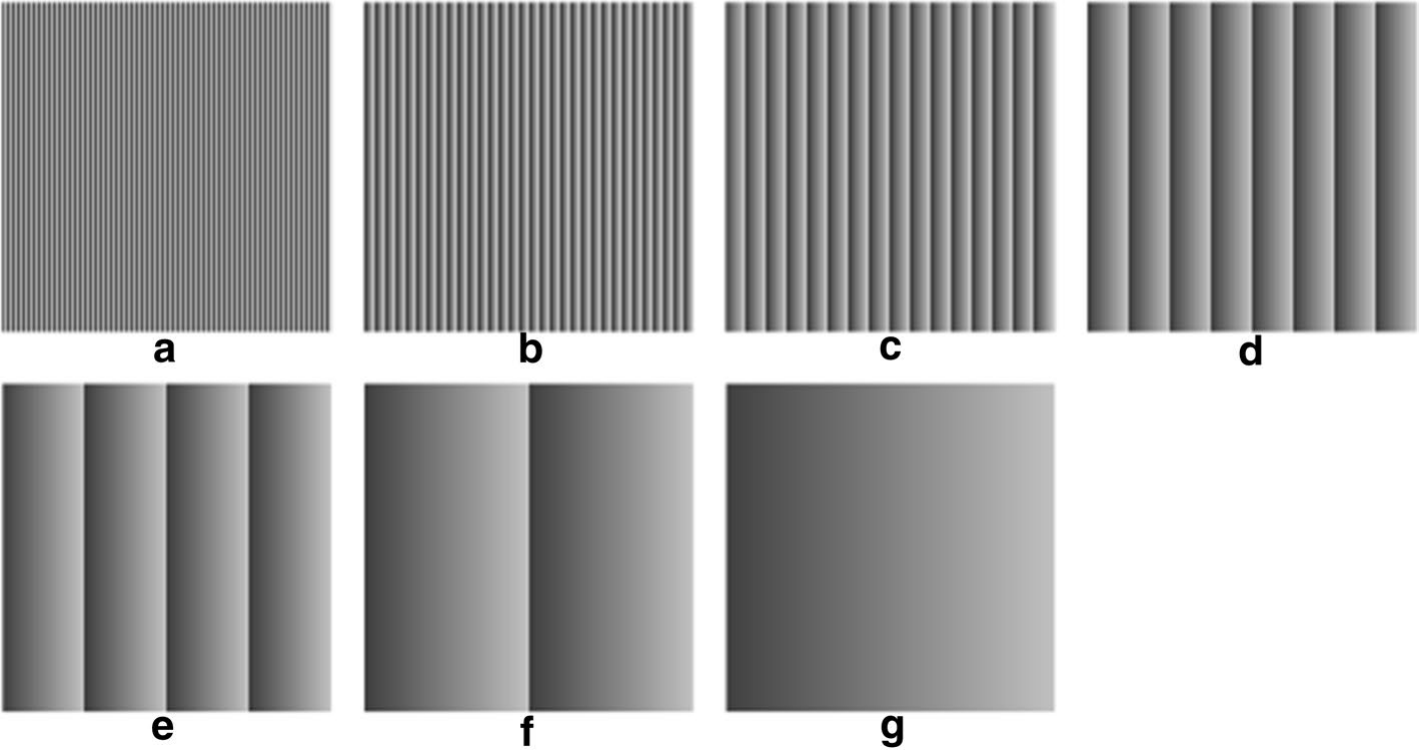
Static frames of the seven synthetic sequences with increasing number of grey levels (2 to 128 in power of 2) equally spaced in the range [64; 192]

The estimation of noise parameters depends on the number and distribution of EVaR points (i.e., the expected value–variance couples) on which the linear regression is performed, i.e., on the number and distribution of grey levels within the scene. For this reason, 14 synthetic sequences were designed to represent static scenes with different number and distribution of grey levels. Each sequence was composed by 100 frames of 128 × 128 pixels represented on 8 bits. The grey levels were assigned to the 128 columns of the scenes in a periodic fashion, from the darkest to the lightest level and then starting again from the darkest one. The first seven sequences, depicted in Fig. 6, included 2 up to 128 grey levels in powers of 2, equally spaced in the interval [64;192], which is 128 wide and centered at the half of the whole representation interval.

**Fig. 7:**
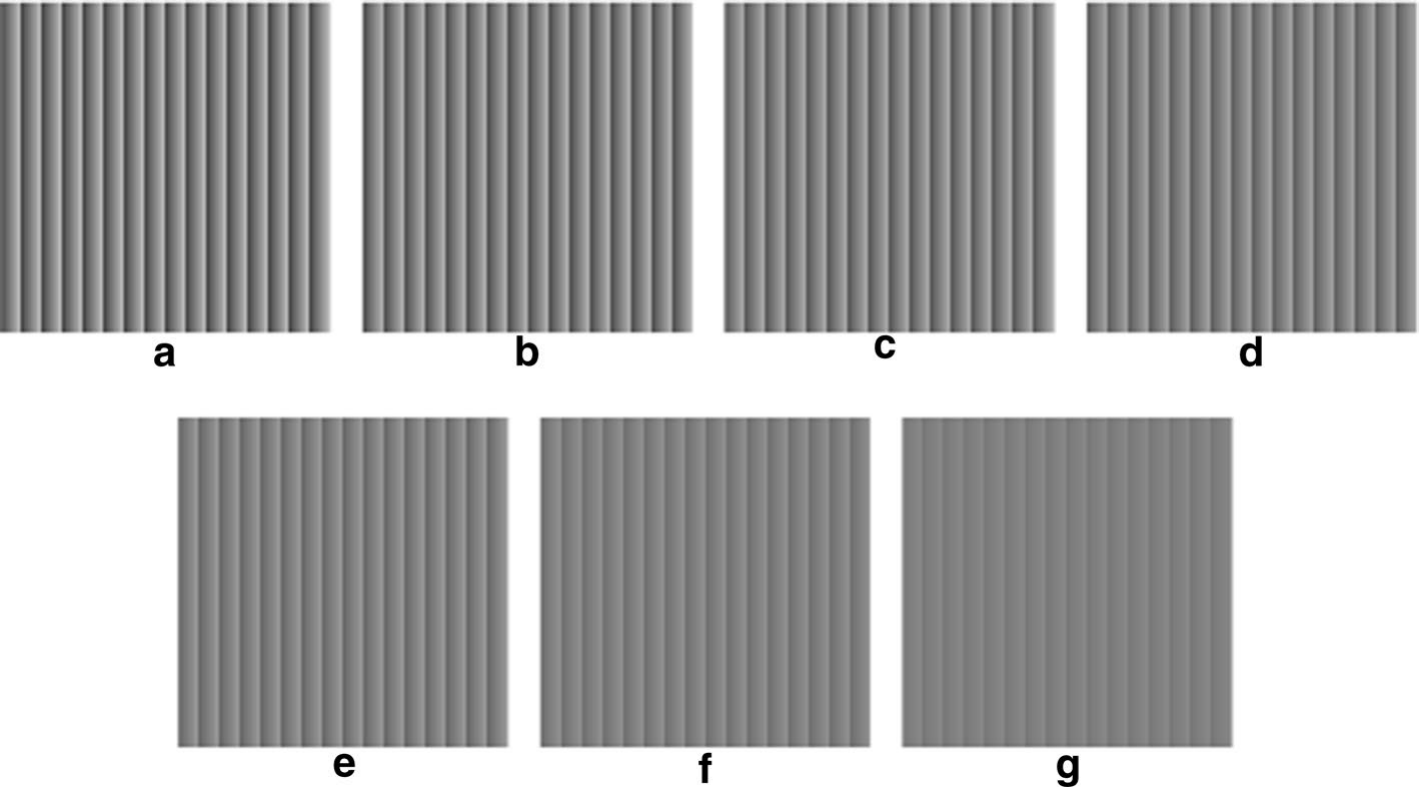
Static frames of the seven synthetic sequences with 8 grey levels equally spaced in narrowing ranges

The other seven sequences, shown in Fig. 7, included 8 grey levels, equally spaced in the intervals described in (5), which are centered at the half of the representation interval and have a decreasing width from 48 down to 16:

5 $$[64 + 8k; 192-8k], \quad k = 1,2, \ldots 7.$$

#### Sequences with moving objects

**Fig. 8:**
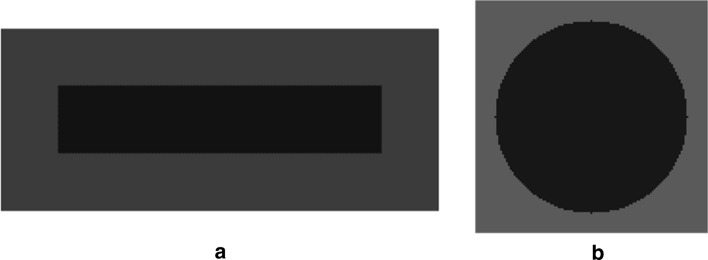
Synthetic sequences with: **a** a moving rectangle and **b** a moving circle, adopted to test the effect of the noise estimation errors on NVCA filtering performances

Considering that the actual concern of denoising is not the mere errors on noise parameters, but rather the reconstruction errors on the processed images, a qualitative and quantitative performance assessment of the noise estimation algorithm analyzed in this study was carried out by comparing the denoising results achieved via the NVCA algorithm with the actual and the estimated noise parameters. To this aim, two synthetic noiseless test sequences were designed, which represented a dark rectangle and a dark circle moving from the left to the right at a speed of 1 pixel per frame over a brighter, uniform background. The noiseless sequences are depicted in Fig. 8.

#### Sequences from X-ray simulator

**Fig. 9:**
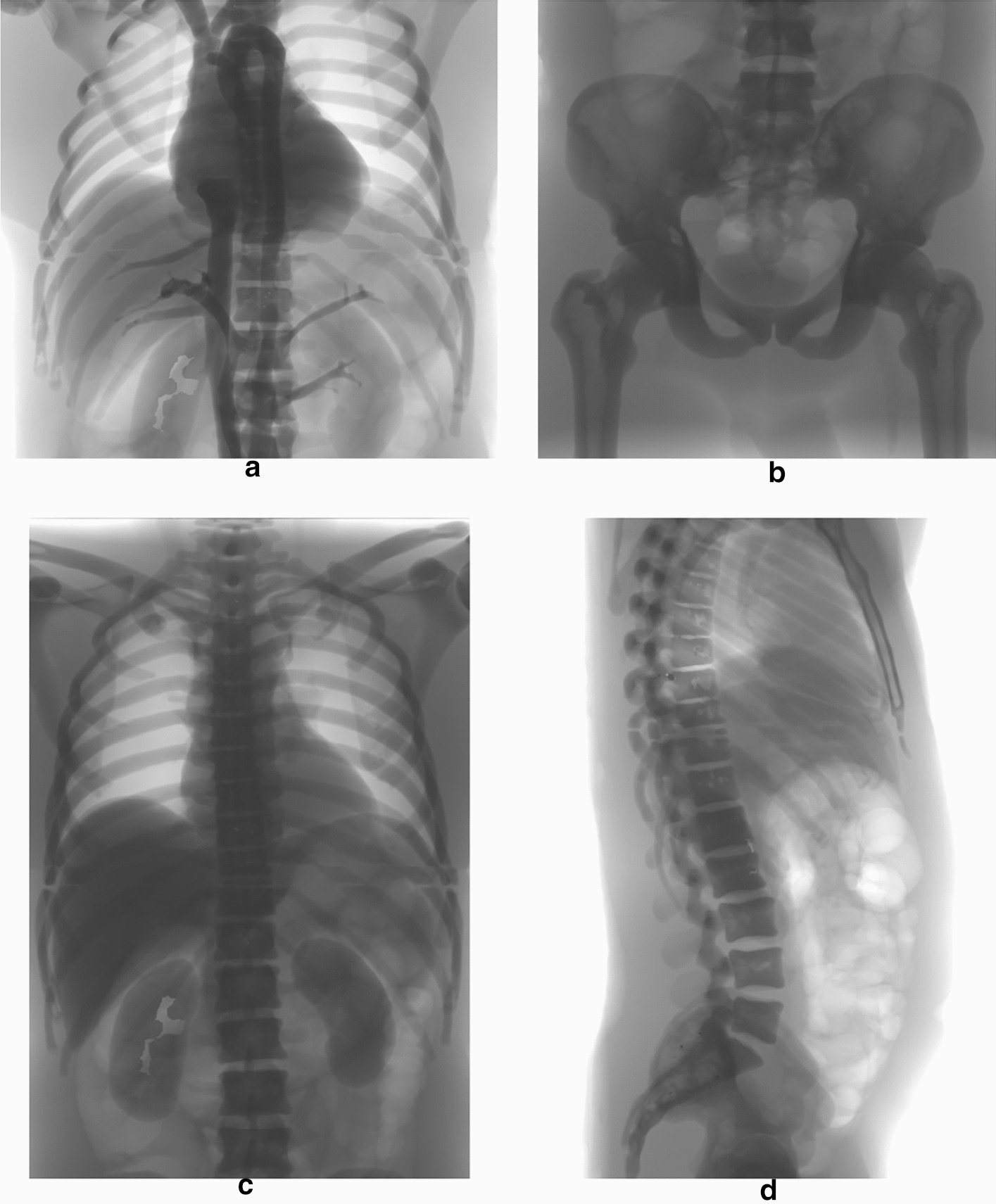
Static frames of the four synthetic sequences devised via the X-ray simulator

The 14 synthetic sequences described in the previous paragraph were characterized by scenes that are very uncommon in medical applications, therefore four additional sequences (see Fig. 9) were produced via an X-ray simulator [44–46], which allowed testing the noise estimation algorithm on scenes with content of clinical relevance, while still having a ground truth to derive quantitative measures for performance assessment.

### Performance analysis

Each of the 14 sequences with variable number of grey levels was corrupted with six different levels of simulated mixed Poisson–Gaussian noise, by using all the combinations of values considered for noise parameters (reported in Table 6). Noise estimation was performed in each of the resulting 84 noisy sequences by considering 4 different number of available frames, i.e., 10, 25, 50, 100. Therefore, a total of 336 noise estimates were actually retrieved (i.e., 56 for each noise level).

**Table 6:** Noise parameters of the noise levels used to corrupt the synthetic scenes

Noise level	*a*	*b*
Level 1	0.5	0
Level 2	1	0
Level 3	2	0
Level 4	0.5	144
Level 5	1	144
Level 6	2	144

**Fig. 10:**
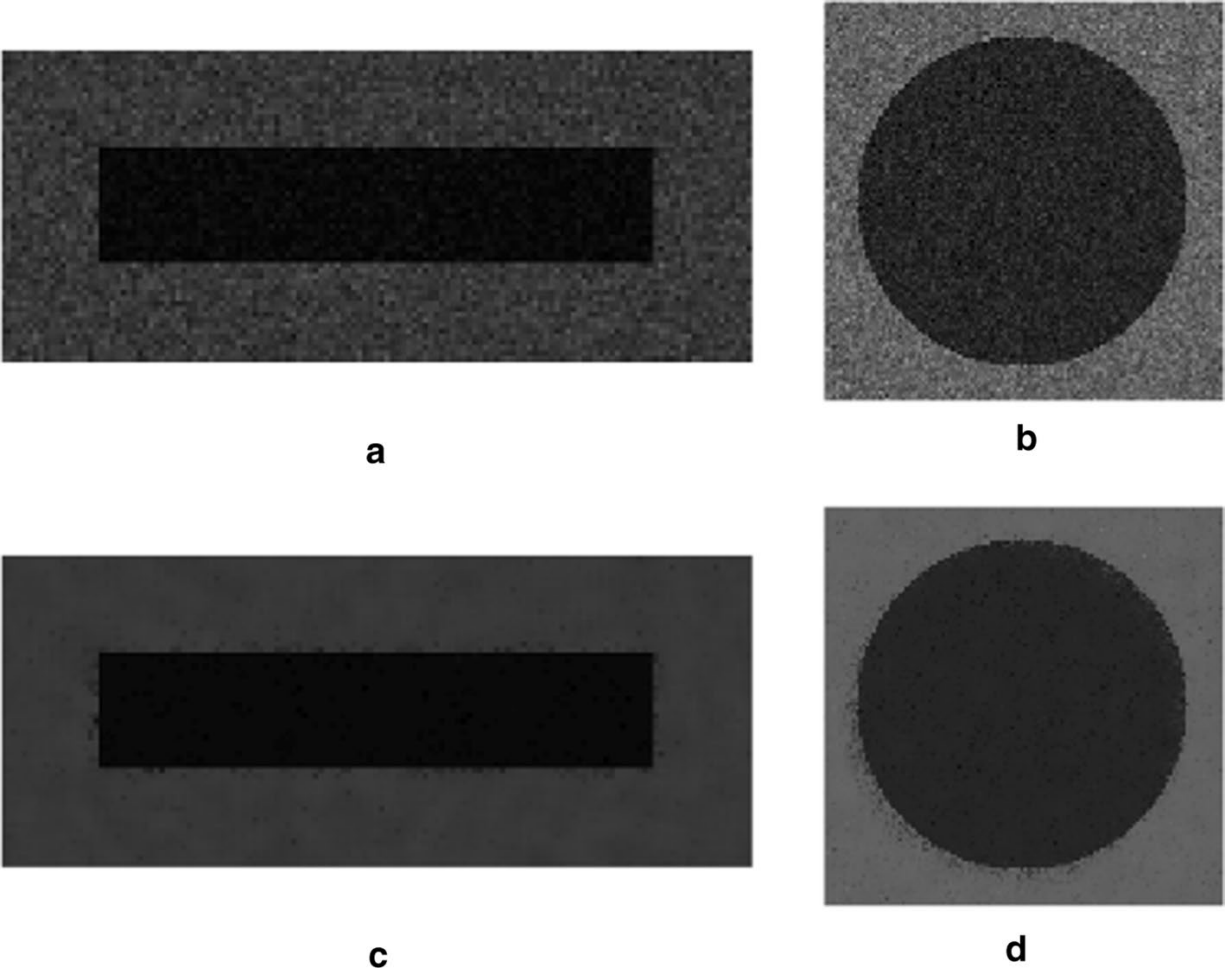
Synthetic sequences with moving objects adopted to test the effect of the noise estimation errors on NVCA filtering performances. In the first row, panels **a**, **b**, the noisy sequences are reported, in which the contrast was set in order to obtain a CNR = 4. In the last row, panels **c**, **d**, the sequences denoised via the NVCA algorithm by using the actual noise parameters are shown

The sequences with moving objects were corrupted with two different levels of mixed Poisson–Gaussian noise, corresponding to level 3 and level 6 reported in Table 6. The contrast in the two noiseless sequences was set to obtain a contrast-to-noise ratio (CNR) of 4 for both sequences. Figure 10 shows a frame from the noisy versions of the sequences with moving objects and the result of NVCA filtering with the actual noise parameters.

**Fig. 11:**
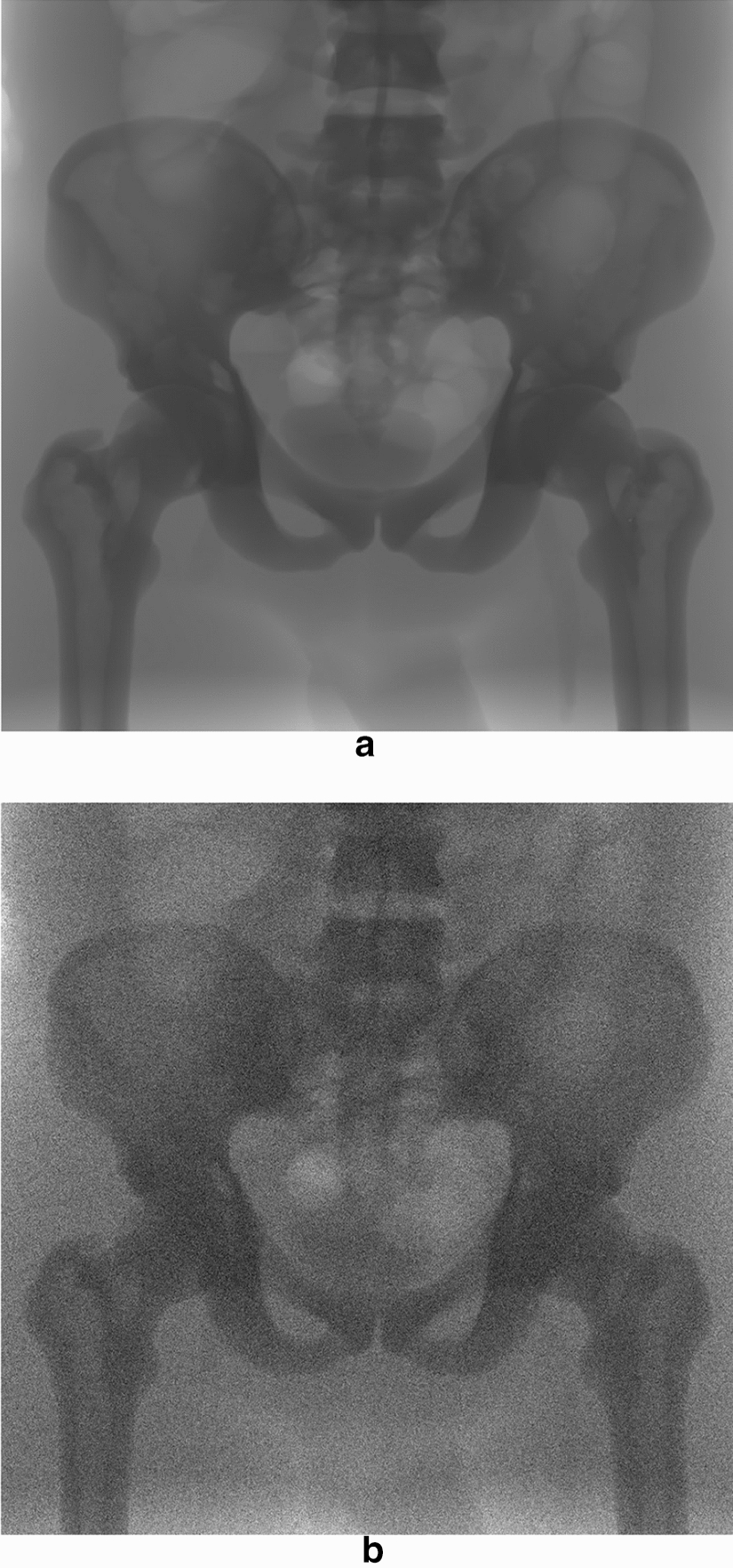
Comparison of noiseless and noisy versions (noise level 6) of the same static frame of sequence #2 from X-ray simulator, depicted in Fig. 9b

The five sequences devised via the X-ray simulator were corrupted with the same noise levels reported in Table 6 (an example of noiseless and noisy versions of a sequence is depicted in Fig. 11), and noise estimation was performed by using 25 frames, which turned out to be the minimum number of frames to retrieve noise parameters with a reasonable accuracy, according to the results reported in paragraph 1 of the “Results” section.

### Real fluoroscopic sequences

**Fig. 12:**
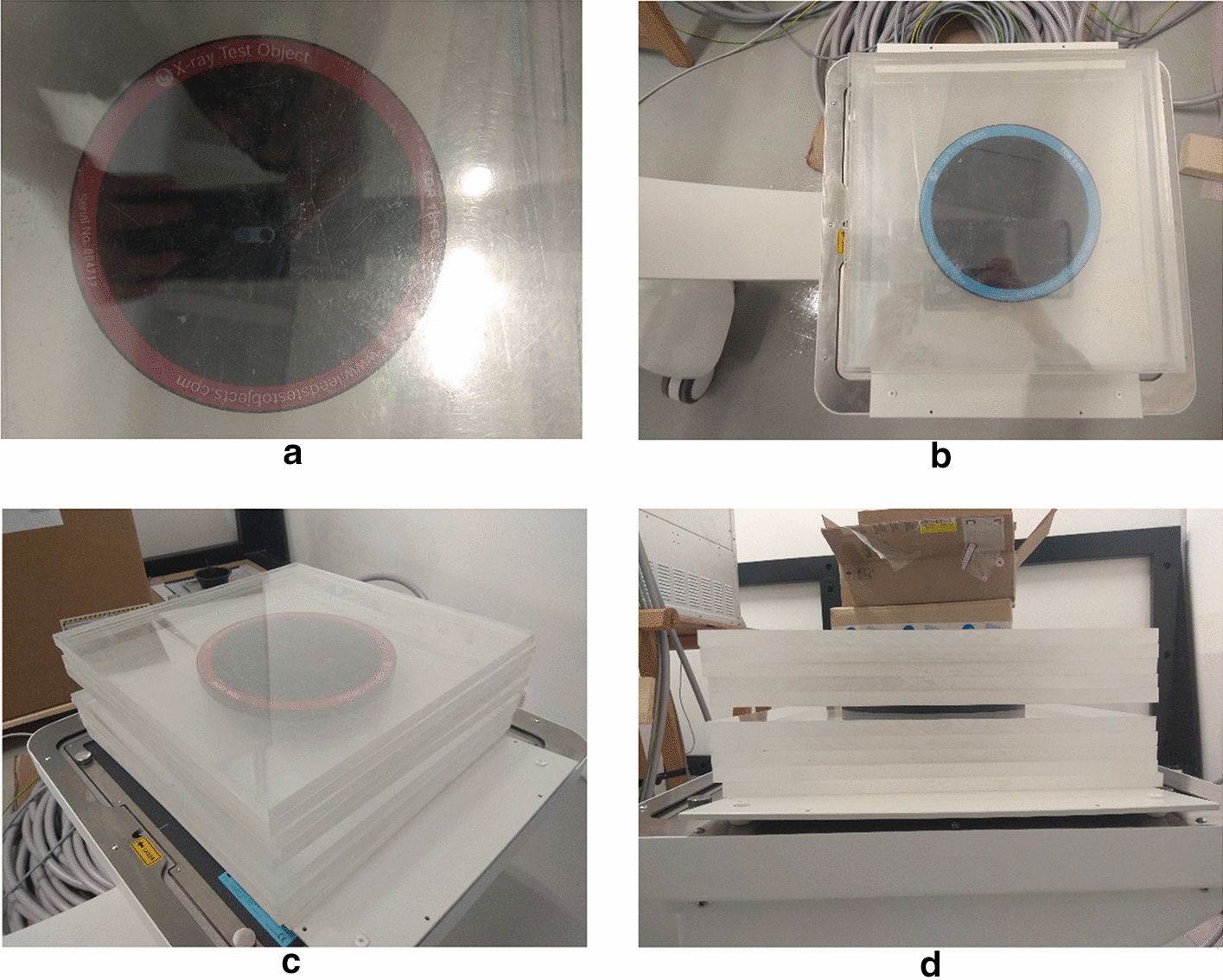
Pictures of the X-ray phantoms with Plexiglass sheets: **a** top view of TOR-18FG; **b** top view of TOR-CDR; **c** side view of TOR-18FG; **d** side view of TOR-CDR

The real fluoroscopic sequences were acquired by imaging two commercial X-ray phantoms, namely TOR-18FG [47] and TOR-CDR [48] (Leeds Test Objects, 7 Becklands Cl, Roecliffe, York YO51 9NR, UK), via a commercial fluoroscopic device (INTERMEDICAL S.r.l. IMD Group, Via E. Fermi 26, 24,050 Grassobbio (BG), Italy). The fluoroscope acquired frames of 1536 × 1536 pixels, represented on 16 bit, with a pulsed protocol at 15 frames per second (fps). Each phantom was placed over an anti-scatter grid, just above the flat panel detector, between two blocks of five Plexiglass square sheets of 25 cm × 25 cm × 1 cm (see Fig. 12), which were used to produce an equivalent Compton scattering noise that would occur when imaging the human body. Five sequences were acquired for each phantom by using the X-ray tube settings reported in Table 7.

**Table 7:** X-ray tube settings used to acquire the real fluoroscopic sequences

X-ray tube setting	Kilovoltage peak (kV)	Current (mA)
#1	40	10
# 2	40	20
# 3	40	30
# 4	40	40
# 5	40	50

The noise estimation algorithm was applied to extract the noise parameters estimates from the ten acquired fluoroscopic sequences. Then, a comparison was carried out between parameters extracted from each couple of sequences acquired with the same X-ray tube settings.

## Supplementary Information

**Additional file 1:Table S1.** Noise parameters estimates extracted from the synthetic sequences with variable number of grey levels. **Table S2.** Errors on noise parameters estimates extracted from the synthetic sequences with variable number of grey levels. All values are expressed as relative errors, except for those reported in blue, which are expressed as absolute errors, since they refer to a null parameter (b = 0). **Table S3.** Noise parameters estimates extracted from the synthetic sequences designed via the X-ray simulator. **Table S4.** Errors on noise parameters estimates extracted from the synthetic sequences designed via the X-ray simulator. All values are expressed as relative errors, except for those reported in blue, which are expressed as absolute errors, since they refer to a null parameter (b = 0).

## Data Availability

All data generated or analyzed during this study are included in this published article, apart from the acquired fluoroscopic sequences. The data are available from Technix S.p.A. (Via Fermi, 45 24050 Grassobbio (BG), Italy), but restrictions apply to the availability of these data, which were used under license for the current study, and so are not publicly available. Data are, however, available from the authors upon reasonable request and with permission of Technix S.p.A.
