# Supplementary material for: Toward a priori noise characterization for real-time edge-aware denoising in fluoroscopic devices
Source: Biomed Eng Online. 2021 Apr 7;20:36. doi: 10.1186/s12938-021-00874-8 (PMC8028787; doi:10.1186/s12938-021-00874-8)
Supplement: Supplementary file 1 — Additional file 1:Table S1. Noise parameters estimates extracted from the synthetic sequences with variable number of grey levels. Table S2. Errors on noise parameters estimates extracted from the synthetic sequences with variable number of grey levels. All values are expressed as relative errors, except for those reported in blue, which are expressed as absolute errors, since they refer to a null parameter (b = 0). Table S3. Noise parameters estimates extracted from the synthetic sequences designed via the X-ray simulator. Table S4. Errors on noise parameters estimates extracted from the synthetic sequences designed via the X-ray simulator. All values are expressed as relative errors, except for those reported in blue, which are expressed as absolute errors, since they refer to a null parameter (b = 0). [file 12938_2021_874_MOESM1_ESM.docx]

Table S1. Noise parameters estimates extracted from the synthetic sequences with variable number of grey levels.

| SEQUENCE | NOISE  LEVEL | NOISE PARAMETERS ESTIMATES | | | | | | | |
| --- | --- | --- | --- | --- | --- | --- | --- | --- | --- |
|  |  | F = 100 | | F = 50 | | F = 25 | | F = 10 | |
|  |  | a | b | a | b | a | b | a | b |
| # 1 | Level 1 | 0.499 | 0.219 | 0.499 | 0.164 | 0.496 | 0.486 | 0.492 | 0.541 |
|  | Level 2 | 1.000 | 0.177 | 0.997 | 0.509 | 0.999 | 0.301 | 0.982 | 1.420 |
|  | Level 3 | 2.002 | 0.000 | 1.997 | 0.423 | 1.990 | 0.890 | 1.979 | 1.611 |
|  | Level 4 | 0.501 | 144.123 | 0.498 | 144.267 | 0.497 | 144.292 | 0.485 | 145.350 |
|  | Level 5 | 0.998 | 144.704 | 0.999 | 144.677 | 1.007 | 144.210 | 0.969 | 148.179 |
|  | Level 6 | 1.982 | 145.831 | 1.980 | 145.990 | 1.972 | 146.625 | 1.991 | 142.812 |
| # 2 | Level 1 | 0.499 | 0.000 | 0.497 | 0.263 | 0.497 | 0.319 | 0.502 | 0.000 |
|  | Level 2 | 0.996 | 0.644 | 1.000 | 0.296 | 1.004 | 0.017 | 1.005 | 0.000 |
|  | Level 3 | 1.991 | 1.026 | 1.986 | 1.454 | 1.977 | 1.961 | 1.948 | 5.245 |
|  | Level 4 | 0.499 | 144.388 | 0.492 | 145.639 | 0.483 | 146.561 | 0.484 | 146.777 |
|  | Level 5 | 1.007 | 143.364 | 1.007 | 142.704 | 1.012 | 142.916 | 0.996 | 145.420 |
|  | Level 6 | 1.972 | 146.909 | 1.966 | 147.741 | 1.956 | 148.271 | 1.951 | 150.376 |
| # 3 | Level 1 | 0.499 | 0.000 | 0.499 | 0.000 | 0.498 | 0.076 | 0.495 | 0.475 |
|  | Level 2 | 1.000 | 0.032 | 0.997 | 0.448 | 0.999 | 0.000 | 1.005 | 0.000 |
|  | Level 3 | 2.003 | 0.000 | 2.003 | 0.000 | 1.982 | 2.372 | 1.937 | 7.186 |
|  | Level 4 | 0.494 | 144.731 | 0.492 | 145.069 | 0.491 | 144.987 | 0.478 | 147.119 |
|  | Level 5 | 0.993 | 144.216 | 0.990 | 144.539 | 0.989 | 145.180 | 1.014 | 142.565 |
|  | Level 6 | 1.995 | 144.514 | 1.992 | 144.470 | 1.965 | 147.347 | 1.900 | 154.883 |
| # 4 | Level 1 | 0.500 | 0.000 | 0.501 | 0.000 | 0.499 | 0.183 | 0.498 | 0.290 |
|  | Level 2 | 0.997 | 0.426 | 0.994 | 0.685 | 0.993 | 0.731 | 0.980 | 2.008 |
|  | Level 3 | 2.003 | 0.000 | 2.001 | 0.000 | 1.996 | 0.427 | 1.967 | 4.308 |
|  | Level 4 | 0.503 | 143.416 | 0.504 | 143.169 | 0.507 | 142.878 | 0.509 | 142.205 |
|  | Level 5 | 0.999 | 144.480 | 0.985 | 145.829 | 0.998 | 144.244 | 0.991 | 144.283 |
|  | Level 6 | 2.007 | 143.594 | 1.992 | 145.409 | 1.993 | 145.278 | 1.956 | 149.775 |
| # 5 | Level 1 | 0.499 | 0.225 | 0.500 | 0.163 | 0.498 | 0.371 | 0.499 | 0.546 |
|  | Level 2 | 0.998 | 0.319 | 1.000 | 0.000 | 0.992 | 0.666 | 0.983 | 1.701 |
|  | Level 3 | 2.002 | 0.000 | 2.001 | 0.000 | 1.999 | 0.000 | 1.988 | 0.799 |
|  | Level 4 | 0.498 | 144.208 | 0.496 | 144.494 | 0.485 | 145.650 | 0.489 | 145.185 |
|  | Level 5 | 0.991 | 145.120 | 0.995 | 144.875 | 0.991 | 144.985 | 0.992 | 143.967 |
|  | Level 6 | 1.996 | 144.303 | 2.000 | 144.076 | 1.965 | 148.333 | 1.946 | 150.476 |
| # 6 | Level 1 | 0.499 | 0.000 | 0.500 | 0.000 | 0.502 | 0.000 | 0.502 | 0.000 |
|  | Level 2 | 0.999 | 0.245 | 0.998 | 0.114 | 0.989 | 1.001 | 0.998 | 0.493 |
|  | Level 3 | 1.985 | 2.175 | 1.978 | 3.152 | 1.979 | 3.007 | 1.954 | 5.703 |
|  | Level 4 | 0.500 | 144.032 | 0.493 | 144.862 | 0.504 | 144.187 | 0.475 | 147.657 |
|  | Level 5 | 1.000 | 144.427 | 1.007 | 143.392 | 1.001 | 143.756 | 1.003 | 143.969 |
|  | Level 6 | 1.967 | 148.324 | 1.962 | 148.507 | 1.948 | 150.805 | 1.939 | 151.995 |
| # 7 | Level 1 | 0.497 | 0.297 | 0.497 | 0.232 | 0.502 | 0.000 | 0.502 | 0.000 |
|  | Level 2 | 1.001 | 0.000 | 1.000 | 0.000 | 0.998 | 0.000 | 0.989 | 0.932 |
|  | Level 3 | 2.005 | 0.000 | 2.005 | 0.000 | 1.991 | 1.350 | 1.998 | 0.000 |
|  | Level 4 | 0.490 | 145.374 | 0.496 | 144.925 | 0.503 | 144.809 | 0.518 | 143.333 |
|  | Level 5 | 1.004 | 143.627 | 1.000 | 144.352 | 1.000 | 144.691 | 0.989 | 144.986 |
|  | Level 6 | 1.994 | 145.828 | 1.992 | 145.552 | 1.966 | 148.917 | 1.981 | 145.708 |
| # 8 | Level 1 | 0.499 | 0.092 | 0.496 | 0.427 | 0.493 | 0.958 | 0.488 | 1.766 |
|  | Level 2 | 0.997 | 0.525 | 1.004 | 0.000 | 1.003 | 0.000 | 1.006 | 0.341 |
|  | Level 3 | 1.984 | 1.572 | 1.980 | 2.232 | 1.974 | 2.874 | 1.956 | 5.122 |
|  | Level 4 | 0.495 | 144.268 | 0.487 | 145.075 | 0.477 | 147.021 | 0.471 | 148.122 |
|  | Level 5 | 0.988 | 145.929 | 1.004 | 144.260 | 0.997 | 144.697 | 0.998 | 145.961 |
|  | Level 6 | 1.959 | 149.049 | 1.957 | 149.624 | 1.959 | 149.799 | 1.914 | 154.707 |
| # 9 | Level 1 | 0.500 | 0.000 | 0.499 | 0.000 | 0.499 | 0.000 | 0.499 | 0.000 |
|  | Level 2 | 0.995 | 0.806 | 0.987 | 1.750 | 0.979 | 2.564 | 0.991 | 1.684 |
|  | Level 3 | 1.982 | 2.547 | 1.975 | 3.152 | 1.987 | 1.219 | 1.963 | 4.332 |
|  | Level 4 | 0.505 | 143.527 | 0.506 | 142.999 | 0.511 | 142.740 | 0.518 | 142.698 |
|  | Level 5 | 1.002 | 143.975 | 0.991 | 145.257 | 0.967 | 147.542 | 0.970 | 147.524 |
|  | Level 6 | 1.985 | 145.971 | 1.960 | 149.439 | 1.979 | 146.578 | 1.982 | 146.331 |
| # 10 | Level 1 | 0.499 | 0.006 | 0.496 | 0.408 | 0.496 | 0.380 | 0.494 | 0.753 |
|  | Level 2 | 0.996 | 0.263 | 0.996 | 0.243 | 0.991 | 0.840 | 0.976 | 2.465 |
|  | Level 3 | 1.995 | 0.862 | 1.987 | 1.832 | 1.961 | 5.898 | 1.902 | 13.168 |
|  | Level 4 | 0.488 | 145.676 | 0.500 | 144.077 | 0.519 | 142.098 | 0.518 | 141.881 |
|  | Level 5 | 0.993 | 145.449 | 0.996 | 144.993 | 0.966 | 148.708 | 0.936 | 152.320 |
|  | Level 6 | 1.997 | 144.294 | 1.981 | 145.680 | 1.957 | 149.669 | 1.818 | 167.430 |
| # 11 | Level 1 | 0.500 | 0.000 | 0.499 | 0.191 | 0.493 | 0.983 | 0.473 | 3.255 |
|  | Level 2 | 0.995 | 0.663 | 1.000 | 0.000 | 0.994 | 0.735 | 0.980 | 2.463 |
|  | Level 3 | 1.988 | 1.764 | 1.966 | 4.574 | 1.966 | 4.751 | 1.901 | 12.557 |
|  | Level 4 | 0.503 | 144.160 | 0.501 | 144.395 | 0.499 | 144.464 | 0.495 | 145.074 |
|  | Level 5 | 1.000 | 143.979 | 1.010 | 142.701 | 1.004 | 143.532 | 0.943 | 153.708 |
|  | Level 6 | 1.968 | 148.911 | 1.917 | 155.485 | 1.890 | 159.302 | 1.832 | 165.283 |
| # 12 | Level 1 | 0.500 | 0.000 | 0.499 | 0.088 | 0.498 | 0.369 | 0.501 | 0.000 |
|  | Level 2 | 1.001 | 0.000 | 1.002 | 0.000 | 1.005 | 0.000 | 0.957 | 5.675 |
|  | Level 3 | 1.979 | 3.193 | 1.956 | 6.100 | 1.944 | 8.350 | 1.805 | 24.896 |
|  | Level 4 | 0.489 | 145.414 | 0.474 | 146.719 | 0.465 | 147.809 | 0.455 | 149.046 |
|  | Level 5 | 0.974 | 147.411 | 1.002 | 143.844 | 0.988 | 145.695 | 0.875 | 159.413 |
|  | Level 6 | 1.928 | 153.771 | 1.903 | 157.066 | 1.873 | 161.648 | 1.642 | 190.281 |
| # 13 | Level 1 | 0.494 | 0.792 | 0.487 | 1.628 | 0.475 | 3.250 | 0.479 | 2.813 |
|  | Level 2 | 0.962 | 4.859 | 0.979 | 2.943 | 0.976 | 3.194 | 0.990 | 1.739 |
|  | Level 3 | 1.928 | 9.451 | 1.890 | 13.995 | 1.801 | 25.274 | 1.612 | 48.691 |
|  | Level 4 | 0.474 | 147.159 | 0.439 | 151.314 | 0.466 | 148.189 | 0.433 | 152.869 |
|  | Level 5 | 1.005 | 143.476 | 0.992 | 145.375 | 0.977 | 147.319 | 0.878 | 160.387 |
|  | Level 6 | 1.952 | 150.488 | 1.914 | 154.792 | 1.834 | 165.616 | 1.505 | 205.340 |
| # 14 | Level 1 | 0.502 | 0.000 | 0.501 | 0.000 | 0.496 | 0.662 | 0.476 | 3.158 |
|  | Level 2 | 0.901 | 12.725 | 0.886 | 14.400 | 0.832 | 21.182 | 0.635 | 46.330 |
|  | Level 3 | 1.779 | 28.759 | 1.754 | 31.707 | 1.425 | 74.866 | 0.984 | 131.379 |
|  | Level 4 | 0.491 | 145.234 | 0.509 | 142.511 | 0.460 | 148.487 | 0.267 | 173.398 |
|  | Level 5 | 0.824 | 166.638 | 0.845 | 163.836 | 0.802 | 169.539 | 0.483 | 210.223 |
|  | Level 6 | 1.652 | 188.843 | 1.540 | 202.970 | 1.183 | 250.552 | 0.622 | 321.903 |

Table S2.. Errors on noise parameters estimates extracted from the synthetic sequences with variable number of grey levels. All values are expressed as relative errors, except for those reported in blue, which are expressed as absolute errors, since they refer to a null parameter (b = 0).

| SEQUENCE | NOISE  LEVEL | ERRORS ON NOISE PARAMETERS ESTIMATES | | | | | | | | | |
| --- | --- | --- | --- | --- | --- | --- | --- | --- | --- | --- | --- |
|  |  | F = 100 | | F = 50 | | F = 25 | | F = 10 | | |  |
|  |  | a | b | a | b | a | b | a | b |  |  |
| # 1 | Level 1 | -0.001622 | 0.2188 | -0.001362 | 0.1636 | -0.007676 | 0.4858 | -0.01546 | 0.5413 |  |  |
|  | Level 2 | 0.000147 | 0.1773 | -0.002913 | 0.5093 | -0.000539 | 0.3010 | -0.01836 | 1.4200 |  |  |
|  | Level 3 | 0.000808 | 0 | -0.001698 | 0.4227 | -0.005084 | 0.8897 | -0.01037 | 1.6110 |  |  |
|  | Level 4 | 0.002798 | 0.000856 | -0.003523 | 0.001852 | -0.005445 | 0.002027 | -0.02912 | 0.009373 |  |  |
|  | Level 5 | -0.002034 | 0.004891 | -0.000668 | 0.004700 | 0.006524 | 0.001460 | -0.03119 | 0.02902 |  |  |
|  | Level 6 | -0.009015 | 0.012720 | -0.01008 | 0.01382 | -0.01420 | 0.01823 | -0.004656 | -0.008250 |  |  |
| # 2 | Level 1 | -0.001200 | 0 | -0.005307 | 0.2627 | -0.006152 | 0.3193 | 0.004515 | 0 |  |  |
|  | Level 2 | -0.003711 | 0.6435 | -0.000341 | 0.2960 | 0.004117 | 0.01663 | 0.005127 | 0 |  |  |
|  | Level 3 | -0.004618 | 1.026 | -0.006989 | 1.454 | -0.01156 | 1.961 | -0.02621 | 5.245 |  |  |
|  | Level 4 | -0.001026 | 0.002692 | -0.01681 | 0.01138 | -0.03331 | 0.01779 | -0.03155 | 0.01929 |  |  |
|  | Level 5 | 0.006921 | -0.004413 | 0.006515 | -0.009002 | 0.01242 | -0.007530 | -0.004158 | 0.009858 |  |  |
|  | Level 6 | -0.01406 | 0.02020 | -0.01708 | 0.02598 | -0.02224 | 0.02966 | -0.02443 | 0.04427 |  |  |
| # 3 | Level 1 | -0.001918 | 0 | -0.002577 | 0 | -0.004570 | 0.07569 | -0.009977 | 0.4749 |  |  |
|  | Level 2 | 0.000387 | 0.03232 | -0.003172 | 0.4481 | -0.001398 | 0 | 0.005147 | 0 |  |  |
|  | Level 3 | 0.001427 | 0 | 0.001465 | 0 | -0.008973 | 2.372 | -0.03152 | 7.186 |  |  |
|  | Level 4 | -0.01189 | 0.005074 | -0.015950 | 0.007423 | -0.01859 | 0.006853 | -0.04303 | 0.021660 |  |  |
|  | Level 5 | -0.006561 | 0.001503 | -0.009869 | 0.003742 | -0.01146 | 0.008194 | 0.01432 | -0.009968 |  |  |
|  | Level 6 | -0.002507 | 0.003573 | -0.004130 | 0.003262 | -0.01725 | 0.02324 | -0.05014 | 0.075570 |  |  |
| # 4 | Level 1 | 0.000441 | 0 | 0.001609 | 0 | -0.002583 | 0.1825 | -0.003028 | 0.290100 |  |  |
|  | Level 2 | -0.002799 | 0.4259 | -0.006336 | 0.684700 | -0.007344 | 0.7314 | -0.02043 | 2.008000 |  |  |
|  | Level 3 | 0.001691 | 0 | 0.000460 | 0 | -0.001950 | 0.4272 | -0.01644 | 4.308000 |  |  |
|  | Level 4 | 0.006652 | -0.004056 | 0.008058 | -0.005771 | 0.01389 | -0.007791 | 0.01802 | -0.012460 |  |  |
|  | Level 5 | -0.000787 | 0.003331 | -0.01491 | 0.01270 | -0.001870 | 0.001695 | -0.008620 | 0.001967 |  |  |
|  | Level 6 | 0.003455 | -0.002816 | -0.004133 | 0.009783 | -0.003350 | 0.008875 | -0.02189 | 0.040100 |  |  |
| # 5 | Level 1 | -0.001011 | 0.225300 | -0.000615 | 0.162700 | -0.003364 | 0.371300 | -0.001869 | 0.5456 |  |  |
|  | Level 2 | -0.002130 | 0.319100 | -0.000221 | 0.000000 | -0.007832 | 0.665900 | -0.017320 | 1.701 |  |  |
|  | Level 3 | 0.001102 | 0.000000 | 0.000465 | 0.000000 | -0.000326 | 0.000000 | -0.006140 | 0.7991 |  |  |
|  | Level 4 | -0.004444 | 0.001447 | -0.007622 | 0.003427 | -0.029570 | 0.011460 | -0.022970 | 0.008231 |  |  |
|  | Level 5 | -0.009281 | 0.007780 | -0.004637 | 0.006079 | -0.008675 | 0.006838 | -0.008394 | -0.000229 |  |  |
|  | Level 6 | -0.002057 | 0.002102 | 0.000003 | 0.000526 | -0.017370 | 0.030090 | -0.026780 | 0.04497 |  |  |
| # 6 | Level 1 | -0.001587 | 0.000000 | 0.000337 | 0 | 0.004174 | 0 | 0.003964 | 0 |  |  |
|  | Level 2 | -0.001316 | 0.245200 | -0.001871 | 0.1138 | -0.010900 | 1.001 | -0.001821 | 0.4925 |  |  |
|  | Level 3 | -0.007512 | 2.175000 | -0.01106 | 3.152 | -0.010280 | 3.007 | -0.02297 | 5.703 |  |  |
|  | Level 4 | -0.000030 | 0.000225 | -0.01429 | 0.005989 | 0.008959 | 0.001299 | -0.05014 | 0.02539 |  |  |
|  | Level 5 | -0.000178 | 0.002966 | 0.006713 | -0.004223 | 0.000955 | -0.001693 | 0.003304 | -0.000215 |  |  |
|  | Level 6 | -0.01662 | 0.03003 | -0.01877 | 0.031300 | -0.025810 | 0.047250 | -0.03048 | 0.05552 |  |  |
| # 7 | Level 1 | -0.005057 | 0.2969 | -0.005013 | 0.2316 | 0.003341 | 0 | 0.004020 | 0 |  |  |
|  | Level 2 | 0.001055 | 0 | 0.000433 | 0 | -0.002254 | 0 | -0.01114 | 0.9321 |  |  |
|  | Level 3 | 0.002579 | 0 | 0.002600 | 0 | -0.004530 | 1.350 | -0.00108 | 0 |  |  |
|  | Level 4 | -0.02035 | 0.009543 | -0.008591 | 0.006422 | 0.006392 | 0.005620 | 0.03530 | -0.004633 |  |  |
|  | Level 5 | 0.003927 | -0.002591 | -0.000288 | 0.002445 | 0.000427 | 0.004796 | -0.01073 | 0.006847 |  |  |
|  | Level 6 | -0.003146 | 0.01269 | -0.003923 | 0.01077 | -0.01676 | 0.034150 | -0.009305 | 0.01186 |  |  |
| # 8 | Level 1 | -0.001993 | 0.091940 | -0.007657 | 0.4271 | -0.01399 | 0.9576 | -0.02450 | 1.766 |  |  |
|  | Level 2 | -0.003294 | 0.525300 | 0.003615 | 0 | 0.002592 | 0 | 0.005880 | 0.3413 |  |  |
|  | Level 3 | -0.007831 | 1.572000 | -0.01023 | 2.232 | -0.01307 | 2.874 | -0.02224 | 5.122 |  |  |
|  | Level 4 | -0.01021 | 0.001860 | -0.02542 | 0.007463 | -0.04521 | 0.02098 | -0.05848 | 0.02863 |  |  |
|  | Level 5 | -0.01153 | 0.013390 | 0.003935 | 0.001808 | -0.003043 | 0.004837 | -0.002356 | 0.01362 |  |  |
|  | Level 6 | -0.02038 | 0.035060 | -0.02153 | 0.03906 | -0.02069 | 0.04027 | -0.04284 | 0.07436 |  |  |
| # 9 | Level 1 | 0.000822 | 0 | -0.001409 | 0 | -0.002329 | 0 | -0.001581 | 0 |  |  |
|  | Level 2 | -0.004964 | 0.8065 | -0.01324 | 1.750 | -0.02094 | 2.564 | -0.008894 | 1.684 |  |  |
|  | Level 3 | -0.008879 | 2.547 | -0.01256 | 3.152 | -0.006424 | 1.219 | -0.01865 | 4.332 |  |  |
|  | Level 4 | 0.01067 | -0.003287 | 0.01282 | -0.006951 | 0.02169 | -0.008748 | 0.03546 | -0.009039 |  |  |
|  | Level 5 | 0.001558 | -0.000175 | -0.009342 | 0.008730 | -0.03297 | 0.02460 | -0.03049 | 0.02447 |  |  |
|  | Level 6 | -0.007353 | 0.01369 | -0.02005 | 0.03777 | -0.01034 | 0.01790 | -0.008994 | 0.01619 |  |  |
| # 10 | Level 1 | -0.001989 | 0.006256 | -0.008282 | 0.4079 | -0.007119 | 0.3799 | -0.01220 | 0.753 |  |  |
|  | Level 2 | -0.003547 | 0.2626 | -0.004008 | 0.2426 | -0.009263 | 0.8396 | -0.02407 | 2.465 |  |  |
|  | Level 3 | -0.002648 | 0.8622 | -0.006260 | 1.832 | -0.01971 | 5.898 | -0.04900 | 13.17 |  |  |
|  | Level 4 | -0.02324 | 0.01164 | 0.000743 | 0.000532 | 0.03717 | -0.01321 | 0.03580 | -0.01471 |  |  |
|  | Level 5 | -0.007359 | 0.01006 | -0.003747 | 0.006894 | -0.03355 | 0.03269 | -0.06375 | 0.05778 |  |  |
|  | Level 6 | -0.001557 | 0.002040 | -0.009616 | 0.01167 | -0.02151 | 0.03937 | -0.09101 | 0.1627 |  |  |
| # 11 | Level 1 | 0.000627 | 0 | -0.001722 | 0.1909 | -0.01487 | 0.9834 | -0.05326 | 3.255 |  |  |
|  | Level 2 | -0.005126 | 0.6625 | -0.000014 | 0 | -0.005635 | 0.7346 | -0.01979 | 2.463 |  |  |
|  | Level 3 | -0.005984 | 1.764 | -0.01681 | 4.5740 | -0.01691 | 4.751 | -0.04937 | 12.56 |  |  |
|  | Level 4 | 0.006719 | 0.001114 | 0.002225 | 0.002745 | -0.002921 | 0.003226 | -0.009365 | 0.007462 |  |  |
|  | Level 5 | -0.000336 | -0.000143 | 0.009847 | -0.009022 | 0.004354 | -0.003249 | -0.05690 | 0.06742 |  |  |
|  | Level 6 | -0.01611 | 0.03410 | -0.04154 | 0.07976 | -0.05512 | 0.1063 | -0.08422 | 0.14780 |  |  |
| # 12 | Level 1 | -0.000121 | 0 | -0.001755 | 0.08846 | -0.004752 | 0.3688 | 0.002222 | 0 |  |  |
|  | Level 2 | 0.001269 | 0 | 0.001948 | 0 | 0.004593 | 0 | -0.04290 | 5.675 |  |  |
|  | Level 3 | -0.01072 | 3.193 | -0.02200 | 6.100 | -0.028020 | 8.350 | -0.09756 | 24.90 |  |  |
|  | Level 4 | -0.02278 | 0.009821 | -0.05286 | 0.01889 | -0.06921 | 0.026450 | -0.08939 | 0.03504 |  |  |
|  | Level 5 | -0.02589 | 0.02369 | 0.002370 | -0.001086 | -0.01179 | 0.011770 | -0.1250 | 0.1070 |  |  |
|  | Level 6 | -0.03576 | 0.06785 | -0.04850 | 0.090730 | -0.06346 | 0.122600 | -0.1791 | 0.3214 |  |  |
| # 13 | Level 1 | -0.01221 | 0.7919 | -0.02520 | 1.628 | -0.05081 | 3.250 | -0.04138 | 2.813 |  |  |
|  | Level 2 | -0.03817 | 4.859 | -0.02115 | 2.943 | -0.02430 | 3.194 | -0.01030 | 1.739 |  |  |
|  | Level 3 | -0.03595 | 9.451 | -0.05524 | 13.99 | -0.09944 | 25.27 | -0.1940 | 48.690 |  |  |
|  | Level 4 | -0.05143 | 0.02194 | -0.1226 | 0.05079 | -0.06853 | 0.02909 | -0.1332 | 0.06159 |  |  |
|  | Level 5 | 0.004782 | -0.003642 | -0.007894 | 0.009551 | -0.02313 | 0.02305 | -0.1220 | 0.1138 |  |  |
|  | Level 6 | -0.02415 | 0.04505 | -0.04312 | 0.07494 | -0.08303 | 0.1501 | -0.2475 | 0.4260 |  |  |
| # 14 | Level 1 | 0.003616 | 0 | 0.002293 | 0 | -0.008299 | 0.6618 | -0.04700 | 3.158 |  |  |
|  | Level 2 | -0.09893 | 12.73 | -0.1135 | 14.40 | -0.1684 | 21.18 | -0.3650 | 46.33 |  |  |
|  | Level 3 | -0.1106 | 28.76 | -0.1228 | 31.71 | -0.2873 | 74.87 | -0.5078 | 131.4 |  |  |
|  | Level 4 | -0.01764 | 0.008571 | 0.01763 | -0.01034 | -0.07998 | 0.03116 | -0.4655 | 0.2042 |  |  |
|  | Level 5 | -0.1762 | 0.1572 | -0.1549 | 0.138 | -0.1980 | 0.1774 | -0.5172 | 0.4599 |  |  |
|  | Level 6 | -0.1741 | 0.3114 | -0.2301 | 0.410 | -0.4087 | 0.7399 | -0.6890 | 1.235 |  |  |

Table S3. Noise parameters estimates extracted from the synthetic sequences designed via the X-ray simulator.

| SEQUENCE | NOISE LEVEL | NOISE PARAMETERS ESTIMATES | |
| --- | --- | --- | --- |
|  |  | F = 25 | |
|  |  | a | b |
| # 1 | Level 1 | 0.500 | 0.060 |
|  | Level 2 | 0.997 | 0.471 |
|  | Level 3 | 1.975 | 3.426 |
|  | Level 4 | 0.498 | 144.398 |
|  | Level 5 | 0.986 | 145.765 |
|  | Level 6 | 1.925 | 153.412 |
| # 2 | Level 1 | 0.495 | 0.475 |
|  | Level 2 | 0.992 | 0.895 |
|  | Level 3 | 1.983 | 1.892 |
|  | Level 4 | 0.486 | 145.463 |
|  | Level 5 | 0.978 | 146.466 |
|  | Level 6 | 1.958 | 148.589 |
| # 3 | Level 1 | 0.497 | 0.321 |
|  | Level 2 | 1.000 | 0.145 |
|  | Level 3 | 1.964 | 4.633 |
|  | Level 4 | 0.496 | 144.592 |
|  | Level 5 | 0.984 | 146.179 |
|  | Level 6 | 1.894 | 156.968 |
| # 4 | Level 1 | 0.497 | 0.217 |
|  | Level 2 | 0.996 | 0.480 |
|  | Level 3 | 1.990 | 1.128 |
|  | Level 4 | 0.492 | 144.824 |
|  | Level 5 | 0.987 | 145.610 |
|  | Level 6 | 1.968 | 147.608 |

Table S4. Errors on noise parameters estimates extracted from the synthetic sequences designed via the X-ray simulator. All values are expressed as relative errors, except for those reported in blue, which are expressed as absolute errors, since they refer to a null parameter (b = 0).

| SEQUENCE | NOISE LEVEL | ERROR ON NOISE PARAMETERS ESTIMATES | |
| --- | --- | --- | --- |
|  |  | F =25 | |
|  |  | a | b |
| # 1 | Level 1 | -0.000727048 | 0.059883648 |
|  | Level 2 | -0.003291033 | 0.470661055 |
|  | Level 3 | -0.012272794 | 3.425821807 |
|  | Level 4 | -0.004674595 | 0.002767017 |
|  | Level 5 | -0.013538244 | 0.012257184 |
|  | Level 6 | -0.037355982 | 0.065361524 |
| # 2 | Level 1 | -0.010395139 | 0.475398655 |
|  | Level 2 | -0.007838176 | 0.894760806 |
|  | Level 3 | -0.008364067 | 1.892067765 |
|  | Level 4 | -0.027658002 | 0.010161948 |
|  | Level 5 | -0.022149186 | 0.017126109 |
|  | Level 6 | -0.021242895 | 0.031867285 |
| # 3 | Level 1 | -0.00529215 | 0.320866941 |
|  | Level 2 | -0.000132407 | 0.145309017 |
|  | Level 3 | -0.017766988 | 4.632563092 |
|  | Level 4 | -0.008127859 | 0.004107655 |
|  | Level 5 | -0.015820705 | 0.015130572 |
|  | Level 6 | -0.053154461 | 0.09005548 |
| # 4 | Level 1 | -0.005063311 | 0.216921236 |
|  | Level 2 | -0.003516649 | 0.479907074 |
|  | Level 3 | -0.004842556 | 1.127681369 |
|  | Level 4 | -0.016299257 | 0.005725547 |
|  | Level 5 | -0.013160495 | 0.0111835 |
|  | Level 6 | -0.01615601 | 0.025058435 |
